# Managing patients with acute and chronic non-specific neck pain: are Danish chiropractors compliant with guidelines?

**DOI:** 10.1186/s12998-017-0148-9

**Published:** 2017-06-30

**Authors:** Simon Sidenius Brockhusen, André Bussières, Simon David French, Henrik Wulff Christensen, Tue Secher Jensen

**Affiliations:** 1 Nordic Institute of Chiropractic and Clinical Biomechanics, Odense, Denmark; 2 School of Physical and Occupational Therapy, Faculty of Medicine, McGill University, Montreal, Canada; 3 Département Chiropratique, Université du Québec à Trois-Rivières, Trois-Rivières, Canada; 4 School of Rehabilitation Therapy, Queen’s University, Kingston, Canada; 5 Medical Department, Spine Centre of Southern Denmark, Middelfart, Denmark; 6 Institute of Regional Health Research, University of Southern Denmark, Odense, Denmark

**Keywords:** Chiropractic, Clinical practice guidelines, Neck-pain, Treatment; management

## Abstract

**Background:**

Non-specific neck pain represents a quarter of all chiropractic patient visits in Denmark. Evidence informed practice can help ensure providers use best available treatment, speed up patient recovery rate and reduce healthcare utilization. It is generally believed that Danish chiropractors treat according to best practice, but we do not know if this is true for management of neck-pain. The objective of this study was to investigate how Danish chiropractors treat patients with acute and chronic non-specific neck pain and determine if management is compliant with recent Canadian guideline recommendations.

**Methods:**

An online survey was sent to 554 members of the Danish chiropractic association. A three-part questionnaire was administered asking participants to: 1) rank the frequency of use of a list of treatment modalities; 2) rank treatment modalities they normally use for acute and chronic non-specific neck pain cases; and 3) provide demographic data. Treatment modalities ranked as “used often” were considered in further analysis and compared to the Canadian Guideline recommendations for neck pain. Chi-squared test was used to investigate differences between treatment and guideline compliance for chronic and acute patients.

**Results:**

A 65% (362/544) response rate was achieved. The sample demographics were representative of a recent Danish study of the entire chiropractic profession. Danish chiropractors use a wide range of treatment modalities, including spinal manipulation, manual therapy, exercises and information/patient education on most of their acute neck pain patients. The use of other treatment modalities and especially exercises was more commonly used with chronic cases. Guideline compliance was 10% for recommendations for acute patients and 43% for chronic patients.

**Conclusions:**

Danish chiropractors use a wide range of treatment options for managing adult patients with acute and chronic non-specific neck-pain. However, there were important differences in treatments chiropractors offered for acute and chronic patients, particularly for the use of exercise therapy, which was mainly reserved for chronic patients. Danish chiropractors’ compliance with guidelines for neck-pain patients was low, but is neither worse nor better than what is seen for other complaints or health disciplines. Our findings suggest a need for active knowledge translation strategies and robust implementation research.

**Electronic supplementary material:**

The online version of this article (doi:10.1186/s12998-017-0148-9) contains supplementary material, which is available to authorized users.

## Background

The estimated annual prevalence of non-specific neck pain is around 35% in Denmark and Scandinavia [1, 2]. In the past 2 weeks alone, 13% of the Danish population (5.6 million) will have experienced major discomfort from the neck and shoulder area [3]. Neck pain results in 4.5 million days of sick leave [4] and annual direct and indirect costs of nearly 430 million USD [4].

Neck pain is considered an episodic condition [5] and people with acute or chronic neck pain seeking treatment usually consult general medical physicians, physiotherapists and chiropractors. In Denmark, 15% of people with neck pain seek chiropractic care [6]. Not surprisingly, the societal and economical impact due to absence of work and treatment costs related to neck-pain are significant [4]. Evidence Informed Practice (EIP) entails using the best available evidence to inform patient care with the aim to speed up the rate of patient recovery and reduce healthcare utilization [7]. The routine use of Clinical Practice Guidelines (CPGs) can help achieve this goal. CPGs are defined by Field and Lohr as “systematically developed statements to assisted practitioner and patient decisions about appropriate health care for specific clinical circumstances” [8].

Release of new CPGs often involves the need for clinicians to change current practice. However, many barriers hinder the uptake of the new knowledge [9]. This underutilization of research evidence is often described as an evidence-practice gap and refers to the difference between what is currently known and what is actually done in clinical practice [10, 11]. The practice of Knowledge Translation (KT) refers to strategies aimed at introducing new knowledge to end-users and organizations to increase implementation [12]. Prior to devoting time and resources in developing and implementing CPG’s, it is prudent to first explore if such evidence-practice gaps exist or if clinicians already practice according to best available evidence. While chiropractors are taught during their academic training to treat patients with neck pain according to the latest and best available evidence, a wide range of treatment modalities are commonly used including, but not limited to: spinal manipulation, mobilization, device-assisted spinal manipulation, education about modifiable lifestyle factors, physical therapy modalities, heat/ice application, massage and other soft tissue therapies such as trigger point therapy, and strengthening and stretching exercises [13]. However, we do not know if all chiropractic neck-pain patients receive the same treatment options or how similar or different the proposed treatment strategies are for managing acute and chronic neck-pain. Further, little is known about whether the chiropractors are compliant with best available evidence for the management of patients with neck pain.

The objective of the current study was to investigate if Danish chiropractors’ management of patients with acute and chronic non-specific neck pain complies with recent Canadian guideline recommendations for the treatment of adults with non-specific neck pain. More specifically, we sought to answer the following research questions:

1. Which treatment modalities do Danish chiropractors use to manage patients with non-specific neck-pain?
2. To what extent does the use of treatment modalities by chiropractors differ for patients with acute or chronic neck-pain?
3. To what extent is the utilization of treatment modalities used by Danish chiropractors compliant with available guideline recommendations?

## Method

### Study design and participants

A cross-sectional online survey was administered between October 30, 2014 and December 20, 2014. All 554 members of the Danish Chiropractic Association (DCA) with a valid email address known by DCA were invited to complete the survey.

### Data collection

#### Guidelines

A PubMed and online search for relevant CPG’s within the past 5 years was carried out and references from each search result were manually reviewed for other relevant guidelines. This revealed only one clinical practice guideline for the chiropractic treatment of adults with non-specific neck pain [14]. The recommendations in this guideline were used to determine guideline compliance by chiropractors. As no effort was made from the DCA to disseminate or implement this particular guideline, we did not expect Danish chiropractors to be aware of the details of the recommendations within the guideline.

#### Questionnaire development and validation

The questionnaire was developed solely for the purpose of this study and was not previously validated. The survey consisted of three parts. The first part included a list of treatment modalities Danish chiropractors commonly use. The list of treatments modalities was informed by an international study investigating treatment preferences of physiotherapists and chiropractors [13]. The list was further discussed among a group of active practitioners (including the first author) and modified to reflect current Danish chiropractic management of patients with neck pain. Included modalities were grouped into one of six treatment groups: 1) High-Velocity Low Amplitude (HVLA) manipulation techniques; 2) other manual techniques; 3) other treatment modalities; 4) exercise therapies; 5) orthoses/supportive devices; or 6) information/patient education. In addition to the manipulation techniques, respondents were asked to indicate the body region or vertebral segment where manipulation would be applied. Some treatment modalities were originally described in English, but where appropriate the name or description was translated into Danish by consensus of the first author and a colleague (CP). Responders were also asked to state if they used any of the treatment type “often”, “occasionally”, “rarely”, “never” or if it was “outside scope of practice” or they would “refer” to someone else for that treatment.

The second part of the questionnaire presented two clinical vignettes, representing a patient with acute neck pain and a patient with chronic neck-pain. For each vignette, chiropractors were asked how they would manage these patients by indicating which treatment group/s they would use and by stating how often they would use each group, using the same response options as above. The two vignettes where developed by the authors based on clinical experience to reflect a ‘typical’ patient presenting with a non-specific neck pain complaint, with no red flags (or serious pathology) and normal cervical spine x-rays.

The last part of the questionnaire included demographics questions (gender, age, graduation year, country of education, employment status and geographic region) adapted from a recent study of the Danish chiropractic profession [15].

### Procedure

The questionnaire was pilot-tested for face and content validity among 8 chiropractors with a range of clinical and research expertise. Written feedback was collected on the comprehensiveness of treatment modalities and the overall understanding of the vignettes and related questions. The questionnaire was modified based on feedback received. An English version of the final questionnaire is available (See Additional file 1).

The questionnaire was administered electronically by the Nordic Institute of Chiropractic and Clinical Biomechanics (NIKKB) and the University of Southern Denmark (SDU) both located in Odense, Denmark, using the licensed SurveyXact software (Rambøll Management Consulting, Aarhus, Denmark). An invitation to participate and a unique questionnaire link was sent out to all Danish chiropractors using e-mail contact information from the DCA. Non-respondents and respondents who had only filled out parts of the questionnaire were sent up to two email reminders after 2 and 3 weeks respectively. Clinicians who indicated they did not wish to participate were not sent reminders.

### Analysis and presentation of data

#### Demographics

Descriptive statistics was used to analyze the demographic characteristics of respondents presented as proportions. The results were compared to a recent study of the Danish Chiropractic profession [15]. In the analysis, the variables age and clinical experience were collapsed into decades.

#### Treatment modalities

The different treatment modalities used by Danish chiropractors were described as count data (actual number and percentage). A given treatment modality was considered to be used if the respondent selected “Often”. A group of modalities as a whole was considered being used by the chiropractor when at least one of the modalities within this group were used “Often”.

Respondents could write additional treatment modalities in plain text if desired. Answers were manually reviewed, and if a similar treatment modality was already stated in the questionnaire, the pre-defined treatment modality was checked accordingly. Any additional treatment options not stated in any of the original questions were coded into new suitable categories for presentation.

#### Vignettes and guideline compliance

The utilization frequency for each modality group used to manage patients with acute and chronic neck-pain was coded as numbers and percentages. Only modality groups stated to be used “Often” were included in further analyses. Differences between the treatment of acute and chronic neck-pain patients were explored using Pearson’s chi-square test and considered significant at the *p* < 0.05 level.

#### Guideline compliance

The Canadian CPG for managing acute and chronic neck-pain recommends that clinicians use multimodal care [14]. This is further elaborated as specific recommendations for modalities and combinations thereof. There are 3 recommendations for acute neck pain, with 2 of them being considered “truly multimodal”, and 6 recommendations for chronic pain, 4 of them considered “truly multimodal” [14]. Guideline compliance for acute neck-pain was defined as any of these 3 combinations: 1) exercise and information/patient education; 2) other manual therapy, exercise and information/patient education; 3) manipulation, other manual therapy, exercise and information/patient education. Options 2 and 3 were considered multimodal. Guideline compliance for chronic neck-pain was defined as any of these 6 combinations: 1) manipulation alone; 2) exercise and information/patient education; 3) exercise and other manual therapy; 4) other manual therapy, exercise and information/patient education; 5) Other manual therapy, other therapy and exercise; 6) manipulation, other manual therapy, other therapy, exercise and information/patient education. Options 3 through 6 were considered multimodal. For both acute and chronic neck-pain descriptive statistics were used to present compliance with any recommendations and compliance to true multimodal treatments. Any differences between these were analyzed using Pearson’s chi-square test and considered significant at the *p* < 0.05 level.

The statistical analyses were carried out using STATA 14 (StataCorp LP, College Station, Texas, USA).

### Ethical considerations

Approvals from The Danish Health Research Ethics Committee System and Danish Data Protection Agency were not required, as no sensitive information were collected regarding racial or ethnic background, political, religious or philosophical conviction, trade union membership information, information about health, sexual or criminal matters, information about significant social problems, or other similar information related to one’s private life and all data were collected anonymously. This is according to Danish legislation stipulated in Act on Research Ethics Review of Health Research Projects, section 14, subsection 1–2 and the Danish Data Protection Act [16].

## Results

### Response-rate and participant demographics

**Fig. 1:**
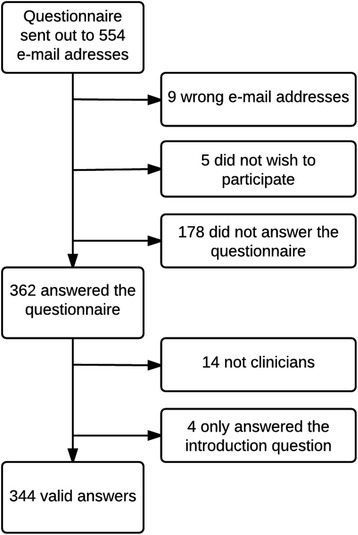
Flowchart demonstrating data collection

Of the 554 DCA members invited to participate, 362 returned their questionnaire (65% response rate) of which 344 completed questionnaires were eligible for analyses (62%). A flowchart of the study, as well as reasons for exclusion in the final analyses can be found in Fig. 1.

The demographics of 344 respondents are summarized in Table 1. Our sample was not statistically different from that of a recent national survey of the Danish chiropractic profession [15].

**Table 1:** Demographic data of 344 chiropractors compared to recent national study

Gender	This Study (%)	Nielsen 2015 (%)	*P*-value^a^
Male	43	43	0.696
Female	50	56	
Missing	6	1	
Age group	This Study (%)	Nielsen 2015 (%)	*P*-value^a^
21–30	9	8	0.996
31–40	28	32	
41–50	25	29	
51–60	22	22	
61–70	8	8	
> 70	<1	<1	
Missing	8	0	
Country of graduation	This Study (%)	Nielsen 2015 (%)	*P*-value^a^
Denmark	44	46	0.305
United Kingdom	20	19	
United States of America	26	29	
Canada	2	2	
Other	0	1	
Missing	7	2	
Years since graduation	This Study (%)	Nielsen 2015 (%)	*P*-value^a^
0–10	26	28	0.821
11–20	15	24	
21–30	18	24	
> 30	11	13	
Missing	30	10	
General employment^a^	This Study (%)	Nielsen 2015 (%)	*P*-value^a^
Clinic owner	58	58	0.942
Private employee	32	30	
Public employee	9	12	
Health ensurance employee	8	6	
Other	3^b^	3	
Missing	7	4	
Region of employment^b^	This Study (%)	Nielsen 2015 (%)	*P*-value^a^
North Denmark Region	7	10	0.962
Central Denmark Region	19	21	
Region of Southern Denmark	28	27	
Region Zealand	16	15	
Capitol region of Denmark	24	26	
Missing	8	1	

^a^The option to give more than one answer was explicitly stated in the questionnaire, ^b^One employed at the Nordic Institute of Chiropractic and Clinical Biomechanics, two external lectors, one PhD student, one on maternity leave, one part time consultant, one unemployed, one censor and one medicine student. Percentages may not total 100% due to rounding

### Utilization of treatment modalities

The different treatment modalities commonly used by Danish chiropractors to manage non-specific neck-pain are shown in Table 2. With the exception of one survey respondent, all clinicians provided patient educational information as part of their management strategy. Almost all chiropractors reported informing their patients of the diagnosis (98%) and monitoring patients’ symptoms (97%). A large proportion also gave information about potential side effects of treatment (80%), and provided some advice on rest/offload (83%) or to keep active (85%). Only 4% of respondents communicated with employers and 8% gave advice on nutritional supplements. Nearly all respondents (97.4%) used HVLA manipulation techniques on patients with neck-pain. The most popular HVLA techniques included Diversified used by 80% of respondents, followed by the Gonstead technique used by over 1/3 of chiropractors. Most respondents (94%) reported that they would manipulate the painful segment, whereas 80% indicated that they would treat the upper cervical and/or thoracic region. Around 20% stated that they would also treat the lumbar and pelvic region, or the extremities, for patients with neck pain.

**Table 2:** Treatment modalities used to manage non-specific neck pain

Manipulation (technique)	Yes 335 (97.4%)	No 9 (2.6%)	Refer	OSP^a^
Diversified	269 (80.3%)	65 (19.4%)	1 (0.3%)	0 (0%)
Gonstead	119 (36.3%)	209 (63.7%)	0 (0%)	0 (0%)
Drop-techniques	58 (19.1%)	238 (78.3%)	1 (0.3%)	7 (2.3%)
Toggle-recoil	39 (12.4%)	263 (83.5%)	2 (0.6%)	11 (3.5%)
Activator	33 (10.5%)	270 (86.3%)	2 (0.6%)	8 (2.6%)
Other manipulation techniques	31 (10.9%)	245 (86.3%)	2 (0.7%)	6 (2.1%)
*Manipulation (region)*	*Yes*	*No*	*Refer*	*OSP* ^*a*^
Segment of pain	311 (94%)	19 (5.7%)	1 (0.3%)	0 (0%)
Upper cervical	257 (79.8%)	64 (19.9%)	1 (0.3%)	0 (0%)
Thoracic	267 (83.2%)	54 (16.8%)	0 (0%)	0 (0%)
Lumbar	64 (20.8%)	243 (79.2%)	0 (0%)	0 (0%)
Pelvis	70 (22.5%)	241 (77.5%)	0 (0%)	0 (0%)
Extremity	56 (18.4%)	248 (81.3%)	1 (0.3%)	0 (0%)
*Other manual therapies*	*Yes 299 (87.9%)*	*No 41 (12.1%)*	*Refer*	*OSP* ^*a*^
Triggerpoint (TrP)	268 (79.8%)	66 (19.6%)	2 (0.6%)	0 (0%)
Instrument Assisted Mobilization (IAM)	8 (2.5%)	289 (91.7%)	4 (1.3%)	14 (4.4%)
Massage	93 (28.5%)	188 (57.7%)	45 (13.8%)	0 (0%)
Massage device	46 (14.1%)	263 (80.4%)	1 (0.3%)	17 (5.2%)
Mechanical Diagnosis Therapy (MDT)	45 (13.9%)	265 (82%)	12 (3.7%)	1 (0.3%)
Mulligan Concept	8 (2.5%)	286 (89.9%)	16 (5%)	8 (2.5%)
Stretch/Muscle energy technique (MET)	81 (24.8%)	242 (74.2%)	3 (0.9%)	0 (0%)
Traction	71 (22%)	246 (76.4%)	2 (0.6%)	3 (0.9%)
Sacro Occipital Technique (SOT)	8 (2.5%)	294 (92.5%)	9 (2.8%)	7 (2.2%)
Craniosacral technique	9 (2.8%)	260 (80.5%)	44 (13.6%)	10 (3.1%)
*Other treatment modalities*	*Yes 208 (62.5%)*	*No 125 (37.5%)*	*Refer*	*OSP* ^*a*^
Transcutan Electric Nerve Stimulation (TENS)	2 (0.6%)	224 (70.7%)	7 (2.2%)	84 (26.5%)
Electromyography (EMG)	0 (0%)	202 (64.1%)	19 (6%)	94 (29.8%)
Short wave	0 (0%)	217 (68.7%)	4 (1.3%)	95 (30.1%)
Laser	23 (7.1%)	235 (72.5%)	13 (4%)	53 (16.4%)
Sonic	0 (0%)	218 (69%)	8 (2.5%)	90 (28.5%)
Ultrasound	1 (0.3%)	208 (66.2%)	26 (8.3%)	79 (25.2%)
Extracorporeal Shockwave Therapy (ESWT)	6 (1.9%)	198 (62.3%)	39 (12.3%)	75 (23.6%)
Heat	26 (8.2%)	272 (86.1%)	1 (0.3%)	17 (5.4%)
Cold	161 (49.4%)	161 (49.4%)	1 (0.3%)	3 (0.9%)
Acupuncture	25 (7.8%)	195 (61.1%)	77 (24.1%)	22 (6.9%)
Dry Needling (DN)	78 (24.3%)	186 (57.9%)	43 (13.4%)	14 (4.4%)
*Exercise*	*Yes 291 (88.2%)*	*No 39 (11.8%)*	*Refer*	*OSP* ^*a*^
Stretch/MET cervical region	170 (52.6%)	146 (45.2%)	7 (2.2%)	0 (0%)
Stretch/MET other body part	82 (26.5%)	221 (71.3%)	7 (2.3%)	0 (0%)
Strength cervical region	154 (48%)	148 (46.1%)	19 (5.9%)	0 (0%)
Strength other body part	86 (27.2%)	211 (66.8%)	19 (6%)	0 (0%)
Motor control	76 (24.1%)	206 (65.2%)	32 (10.1%)	2 (0.6%)
Stability	125 (38.8%)	165 (51.2%)	32 (9.9%)	0 (0%)
Cardio-vascular	40 (12.8%)	241 (77%)	29 (9.3%)	3 (1%)
MDT/McKenzie	89 (27.9%)	214 (67.1%)	16 (5%)	0 (0%)
General physical activity	247 (77.2%)	62 (19.4%)	11 (3.4%)	0 (0%)
*Orthoses*	*Yes 60 (18.2%)*	*No 269 (81.8%)*	*Refer*	*OSP* ^*a*^
Collar	4 (1.2%)	309 (95.4%)	2 (0.6%)	9 (2.8%)
Pillow	36 (11.1%)	280 (86.4%)	3 (0.9%)	5 (1.5%)
Tape	11 (3.4%)	277 (86.3%)	28 (8.7%)	5 (1.6%)
Inserts	8 (2.5%)	279 (87.2%)	27 (8.4%)	6 (1.9%)
Other assistive devices	13 (4.2%)	261 (85%)	23 (7.5%)	10 (3.3%)
*Information/patient education*	*Yes 332 (99.7%)*	*No 1 (0.3%)*	*Refer*	*OSP* ^*a*^
Diagnosis	326 (98.2%)	6 (1.8%)	0 (0%)	0 (0%)
Sideeffects	261 (79.1%)	69 (20.9%)	0 (0%)	0 (0%)
Monitor symptoms	321 (97.3%)	9 (2.7%)	0 (0%)	0 (0%)
Rest/offload	275 (83.3%)	55 (16.7%)	0 (0%)	0 (0%)
Active lifestyle	280 (84.6%)	51 (15.4%)	0 (0%)	0 (0%)
Diet and smoking	68 (21.1%)	254 (78.6%)	1 (0.3%)	0 (0%)
Supplements	27 (8.4%)	293 (91%)	2 (0.6%)	0 (0%)
Ergonomics	200 (60.6%)	128 (38.8%)	2 (0.6%)	0 (0%)
Work/job function	229 (69%)	103 (31%)	0 (0%)	0 (0%)
Free time	233 (70.4%)	98 (29.6%)	0 (0%)	0 (0%)
Employer communication	12 (3.7%)	311 (96%)	1 (0.3%)	0 (0%)
GP communication	88 (26.8%)	240 (73.2%)	0 (0%)	0 (0%)
*Other*	*Yes*	*No*	*Refer*	*OSP* ^*a*^
Modalities not included in questionnaire	41 (12.4%)	289 (87.6%)	0 (0%)	0 (0%)

^a^OSP = Outside Scope of Practice

Other manual therapies used by 88% included trigger-point therapy, massage, stretching and traction. Other treatment modalities were used by 62% of chiropractors, consisting primarily of Cold application and Dry Needling. Most chiropractors prescribed exercise as part of their treatment (88%) with general physical activity being recommended by 3 out of 4 respondents and stretching exercises for the neck recommended by over half of clinicians. Also strengthening of the neck musculature (48%) and stability exercises (39%) were commonly prescribed. Only 18% recommended the use of orthoses or supportive devices, with pillows (11%) and tape (3%) being most popular.

Of the 59 participants stating that they used treatment modalities not previously listed, 18 were coded within pre-defined categories, leaving 41 new treatment options. These were grouped into the following 14 categories: Non-manipulative neurological stimulation (e.g. Z-health exercises), fascia release/Active Release Technique (ART), advice on over the counter painkillers, breathing drills, NeuroImpulse Protocol (NIP), stress management and mindfulness, articulation/mobilization, reflex locomotion/Dynamic Neuromuscular Stabilization (DNS), treatment of the temporomandibular joint or skull, reflexology, Posture Pumps/Dakota traction, Applied Kinesiology (AK), referral to hypnosis or psychologist, Primal Reflex Release Technique (PRRT) and the Mensendieck system.

### Treatment of typical patients with acute or chronic neck-pain

Almost every Danish chiropractor indicated they would use manipulation therapy and information/patient education as part of their treatment approach for both acute and chronic neck-pain patients (over 90% in both cases), whereas 2/3 would also use other types of manual therapy. No statistically significant difference was found between the management of acute and chronic patients for both manipulation, information/patient education and manual therapies. There was a significant difference in treatment offered between acute and chronic neck pain patients, with around 1/3 of respondents saying they would use “other treatment modalities” for acute neck-pain patients, whereas 46% would use these for chronic neck-pain patients (*p* = 0.004). Only 19% reported they would recommend exercise therapy for acute cases compared with 63% for chronic neck-pain patients (*p* < 0.001). Very few chiropractors indicated that they would use orthoses or other supportive devices for when treating neck-pain patients, regardless of symptom duration. The detailed usage of each modality groups for both acute and chronic patients can be found in Table 3.

**Table 3:** Usage of modality-groups for treatment of typical patients with acute and chronic non specific neck-pain

	Acute						Chronic						
	Used	Not used					Used	Not used					
Treatment usage	Often	Occ.*	Rarely	Never	Refer	OSP**	Often	Occ*	Rarely	Never	Refer	OSP**	*p*-value^a^
Manipulation	306 (93,9%)	13 (4%)	3 (0,9%)	3 (0,9%)	1 (0,3%)	0 (0%)	288 (91,1%)	21 (6,6%)	3 (0,9%)	3 (0,9%)	1 (0,3%)	0 (0%)	0,189
Other manual therapies	199 (64,8%)	72 (23,5%)	28 (9,1%)	6 (2%)	2 (0,7%)	0 (0%)	204 (66,7%)	76 (24,8%)	17 (5,6%)	8 (2,6%)	1 (0,3%)	0 (0%)	0,630
Other treatment modalities	101 (34,5%)	87 (29,7%)	79 (27%)	21 (7,2%)	5 (1,7%)	0 (0%)	138 (46%)	107 (35,7%)	48 (16%)	5 (1,7%)	2 (0,7%)	0 (0%)	0,004*
Exercise	56 (19,1%)	91 (31,1%)	109 (37,2%)	32 (10,9%)	3 (1%)	2 (0,7%)	196 (62,8%)	82 (26,3%)	24 (7,7%)	3 (1%)	4 (1,3%)	3 (1%)	<0,001*
Orthoses	1 (0,4%)	20 (7,1%)	79 (28,1%)	181 (64,4%)	0 (0%)	0 (0%)	13 (4,5%)	48 (16,7%)	90 (31,3%)	131 (45,5%)	4 (1,4%)	2 (0,7%)	0,001*
Information/patient education	302 (94,4%)	15 (4,7%)	2 (0,6%)	0 (0%)	1 (0,3%)	0 (0%)	309 (97,5%)	6 (1,9%)	0 (0%)	2 (0,6%)	0 (0%)	0 (0%)	0,189

*Occacionally, **OSP = Outside Scope of Practice

^a^Differences in usage of treatment-groups between acute and chronic patients, tested with chi-squared test, *significant at *p* < 0.05 level. Percentages may not total 100% due to rounding

### Guideline compliance

The guideline compliance for both the acute and chronic recommendations are shown in Table 4. For acute neck-pain, only 10% of survey respondents were compliant with the multimodal care recommendation, whereas 43% were guideline multimodal compliant for chronic neck pain patients. There was a statistically significant higher compliance with recommendations for chronic neck-pain when looking at any compliance (*p* < 0.001) and compliance to multimodal care (*p* < 0.001), as compared to acute neck-pain.

**Table 4:** Compliance with guideline recommendations for acute and chronic non-specific neck pain

Acute			Chronic			
Reccomendation	Compliance	Non-Compliance	Reccomendation	Compliance	Non-Compliance	
#1 Exercise + Information	55 (16,8%)	273 (83,2%)	#1 Manipulation	288 (90,3%)	31 (9,7%)	
#2 Other manual + Exercise + Information	34 (10,4%)	294 (89,6%)	#2 Exercise + Information	195 (61,1%)	124 (38,9%)	
#3 Manipulation + Other manual + Exercise + Information	30 (9,1%)	298 (90,9%)	#3 Exercise + Other manual	138 (43,3%)	181 (56,7%)	
			#4 Other manual + Exercise + Information	137 (42,9%)	182 (57,1%)	
			#5 Other manual + Other tx + Exercise	71 (22,3%)	248 (77,7%)	
			#6 Manipulation + Other manual + Other tx + Exercise + Information	63 (19,7%)	256 (80,3%)	
General compliance	Compliance	Non-Compliance	General compliance	Compliance	Non-Compliance	*P*-value
Overall	55 (16,8%)	273 (83,2%)	Overall	309 (96,9%)	10 (3,1%)	<0.001^a^
Multimodal	34 (10,4%)	294 (89,6%)	Multimodal	138 (43,3%)	181 (56,7%)	<0.001^a^

^a^Differences in compliance between recommendations for acute and chronic patients, tested with chi-squared test, considered significant at *p* < 0.05 level

## Discussion

To the best of our knowledge this is the first study to investigate how Danish chiropractors treat patients with acute and chronic non-specific neck-pain and to determine chiropractors’ compliance with recent published guidelines. All chiropractors reported that they inform their patients about the clinical diagnosis and monitored patients’ symptoms as part of their usual management. Most would advise their patients undertake or keep an active lifestyle or said they delivered advice on rest/offloading. Our results suggest that Danish chiropractors use a wide range of treatment modalities including, but not limited to, spinal manipulation, trigger point therapy, massage, passive stretching, cold application, dry needling and various exercises. Orthoses or other supportive devices were rarely used.

The chiropractors use of manipulation, other manual therapies or information/patient education did not differ between acute and chronic non-specific neck-pain. However the use of other treatment modalities including orthoses and especially exercise was far more common for chronic neck pain patients. The compliance with guideline recommendations was very low for the treatment of acute neck pain (17% for any compliance and 10% for multimodal), which was significantly lower than compliance with the recommendations for chronic patients (97% and 43% respectively).

### Treatment modalities

HVLA-manipulation techniques appears to be utilized by almost every Danish chiropractor. This is not surprising as these techniques are seen as core treatments of the profession [17]. The high use of information/patient education, other manual therapies, exercise and other therapies do however indicate, that HVLA-manipulation is only a part of the toolbox that Danish chiropractors use in their management of neck pain patients. Our findings are in line with the study by Nielsen et al. [15] who also found that HVLA-manipulation is used by nearly all practicing Danish chiropractors (98%), although their study did not specifically focus on neck pain. Another study investigating treatment preferences for neck pain patients between chiropractors and physiotherapists in 19 countries found spinal manipulation to be used commonly by 56% and occasionally by 32% across professions, with a statistically significant higher use by chiropractors [13].

The Diversified technique was the most commonly used (80,3%) type of manipulation technique. This finding is supported by other studies showing ‘Diversified’ technique in general being utilized by up to 93% of chiropractors in Belgium [18] and for neck pain patients in particular by up to 78% on the initial visit [19]. We found mechanical-assisted (Activator) manipulative therapy to be used by only 10% of Danish chiropractors, yet other studies have found this to be used by 20–48% [15, 18, 19]. A reason for this could be the high number of Danish educated chiropractors in our study, where Activator methods only represent a small part of the curriculum. The use of other HVLA techniques was consistent with findings in another study, except we found that Danish chiropractors tend to use the Gonstead technique more often (30%) compared with Belgian chiropractors (11–20%) [18, 19]. Most Danish chiropractors (94%) would address the area of pain, with around 80% treating upper cervical or thoracic spine and around 20% treating lumbar spine, pelvis or extremities; this is consistent with findings by Rubinstein et al. [19]. The use of HVLA techniques in regions other than the symptomatic area, i.e. cervical spine, is interesting, as the guideline provides no recommendations for HVLA-manipulation of other body regions when treating neck pain patients, neither acute nor chronic [14].

The use of soft tissue techniques seems to be higher in our study compared to other studies related to treatment of neck-pain [19], but similar to or lower than other studies looking at the chiropractic profession in general [15] or when compared to physiotherapists [13]. This is possibly because of the time involved in delivering soft tissue therapy considering that Danish chiropractors schedule patients on regular visits with an average duration of 6–15 min [15]. Interestingly, other treatment modalities such as acupuncture or dry needling and laser therapy were less often used for neck pain patients in our study compared to their use in the study by Nielsen et al. for general management of chiropractic patients [15]. This may indicate that Danish chiropractors find these modalities less suitable to treat neck-pain or lack confidence to use it with this type of patients.

Half of the Danish chiropractors reported that they would use cold application and less than 10% would use heat therapy. These findings contrast with other studies, indicating thermal agents (ice or heat) are used by up to 75% of chiropractors and physiotherapists [13, 18], but compare favorably with studies exploring their use on neck pain patients specifically with less than 20% use on the first visits [19]. The widespread use of cold-therapy is noteworthy considering that there is little scientific evidence to support its use for reducing musculoskeletal pain [20–22].

Almost 90% indicated that they routinely prescribed exercise therapy as part of their management for neck-pain patients. This is consistent with other studies showing that 81–98% chiropractors commonly recommend exercise therapy [13, 15, 18, 23]. However Freburger et al. [24] conducted a survey of chronic neck and back pain patients who have consulted either a physiotherapist, a physician or a chiropractor in the past year, asking whether they were prescribed exercise and by whom. Out of 648 patients only 48% was prescribed exercises and out of those only 21% were from chiropractors. In total 33% of patients who saw a chiropractor were prescribed exercises.

Our finding indicates that almost every respondent use information/patient education as part of their management of neck pain patients, chronic and acute. Therefore this can be considered one of the core services provided by Danish chiropractors. The high utilization of information/patient education is in line with what is reported for chiropractors in North America by Coulter et al. [23]. A large study investigating the use of psychological and patient education for neck pain patients across 19 countries, including Denmark, finds the use of patient education among physical therapists and chiropractors to be 95% [25].

### Acute and chronic patients and guideline compliance

We found the use of other treatment modalities such as orthoses/supportive devices and exercise to be significantly higher with chronic patients than for acute patients. This is consistent with findings by Carlesso et al. [13], who found that chronic neck pain patients more often would be treated with exercise, and that respondents usually used a wider range of interventions in management of chronic patients. On the other hand, we found that guideline compliance with ‘any recommendation’ was low for acute patients (17%) but high for chronic patients (97%). This is primarily related to the relative low use of exercise with acute patients in our study, and the fact that manipulation as a single treatment is recommended for chronic patients [14]. For true multimodal treatment, which is the overall recommendation of the guideline [14], the percentage was quite low for both acute (10%) and chronic (43%) patients. But taking into consideration that we did not expect Danish chiropractors to be aware of this CPG, a compliance ranging between 10%–43% may be considered fair. A recent review by Sutton et al. supports the use of multimodal care for the treatment of neck pain and associated disorders [26].

Even though 88% of respondents stated that they often use exercises as part of their management of neck-pain patients, exercise therapy appears to be primarily reserved for chronic patients. We do not know the reason for this distinct difference, but one speculation could be, that chronic patients might have tried simpler approaches without the expected results, and therefore invite to, or even demand, the use of more modalities, including exercise therapy. Another reason could be, that acute patients might experience satisfying outcomes without the use of exercises, and neither the chiropractor nor patient might feel the need to incorporate this into the management, even though it might provide quicker or better results. The apparent low use of exercise with acute patients could also be because some chiropractors might not consider certain management strategies as exercise, such as MDT or stretching.

Evidence Informed Practice (EIP) consists of three sources of knowledge: 1) research evidence; 2) clinical expertise/clinical reasoning, and 3) patient’s preferences and values [27]. When the available evidence is limited (weak-moderate recommendations based on low-moderate quality evidence exist), such as for the management of neck pain, the observed discordance between guideline recommendations and real life clinical practice may reflect that the other two sources of evidence in the EIP model play a larger role when practitioners and patients agree on management strategies.

While few studies have investigated guideline adherence for the management of neck pain, the majority of studies investigating guidelines in general and for low back pain specifically have shown low to moderate adherence across health care professions [28–30]. When this survey was conducted, only a single guideline for chiropractic treatment of neck pain was available. We are aware that this guideline has several limitations; 1) narrow timespan of research included (from 2004 to 2011); 2) recommendations for acute neck pain is based on only 7 studies, whereas the recommendations for chronic neck pain is based on 31 studies; 3) some of the included studies are based on relatively small group sizes; 4) the recommendations are summarized and presented in a very rigid fashion with the possible misinterpretation of the original research results. Ng et al. [31] have compared the quality of this guideline using the Appraisal of Guidelines, Research and Evaluation II (AGREE II) [32] instrument and 2 appraisers both agreed on recommending use with some modifications. They found an average Appraisal Score of 4.7 and an Overall Assessment of 5.0 on a 7-point scale. With Stakeholder Involvement and Editorial Independence as the primary limiting domains. All taken into consideration, this guideline was still considered the best benchmark at the time of the survey. Just recently the Ontario Protocol for Traffic Injury Management (OPTIMa) collaboration published a guideline for management of neck pain and associated disorders [33]. Even though not entirely comparable to the guideline used in this study, as the OPTIMa guideline subgroups neck pain patients differently, a multimodal approach consisting of manual therapy, exercise and education is the main recommendation for grade 1 and 2 neck pain associated disorders [26, 33]. In addition, the Danish Health Authority have recently developed a new National Clinical Guideline for non-surgical management of recent onset neck-pain [34]. These guidelines recommend verbal reassuring information and recommend against the use of written information. Furthermore there are recommendations for exercise and manual therapy (SMT) in addition to other treatment and preferably in combination. Additionally there are recommendation for acupuncture in addition to other treatments and recommendation against the use of massage therapy. The essence is multimodal care with focus on exercise and manual therapy. Considering this, it seems unlikely that the results of our study would have been much different if compared to these new guidelines.

### Strengths and limitations

Despite the satisfying response rate and representativeness of our sample with the Danish chiropractic profession, this survey has a number of limitations. First, our study was cross-sectional design, and therefore only presents a here and now perspective of management trends. Second, we found only a single clinical practice guideline published at the time of the survey, and with Danish chiropractors not being the main audience, this of course limits our results, as we are dependent of the quality of recommendations in this guideline. The rigid fashion of the recommendations and our resulting strict definition of compliance made it harder for our respondents to be guideline compliant. This is especially true for acute neck-pain where only 2 out of 3 recommendations was considered multimodal, both requiring a combinations of 3 or 4 modalities to meet compliance. Had the recommendations been less rigid, our compliance rate would have been much higher. Third, the survey was carried out after the publication of the clinical practice guidelines. To truly uncover an evidence-practice gap the study should have been executed before publication of the guideline. It could be argued, however, that publication alone is not an effective implementation strategy [35], and that the guidelines has not been actively or passively disseminated to the population of Danish chiropractors or patients. However our survey did not include questions to uncover Danish chiropractors level of awareness of the Canadian CPG recommendations. This is considered a strength as we did not want to bias our respondents. While it is possible that some clinicians were familiar with the guidelines, the low adherence rates suggest it is unlikely that this influenced our results. Fourth, we do not know if non-responders are similar or different than the survey responders. The high response rate, the wide diversity of respondents’ characteristics and similar demographic characteristics with a recent national survey suggests that our sample is likely representative of the general Danish chiropractic population. Fifth, the length of the questionnaire and the repetitive nature of the questions may have been a limiting factor. Listing 59 modalities back-to-back and asking respondents to consider each of them in a similar fashion might result in respondent fatigue, leading to participants engaging in “straight-line” responding or dropping answers or the questionnaire all together. As none of the questions were mandatory this could mean that some of the missing answers should actually be considered as “no” instead. However, the number of missing responses was low. As the questions about demographic characteristics was placed last, this did mean that if people decided to skip the questionnaire anywhere through the first 2 parts we do not know anything about them. This was a conscious choice, as we were aware of the risk of questionnaire fatigue and found it more important to get quality data on treatment modalities than demographics. Sixth, the clinical vignettes aimed to mimic acute and chronic patients may have lead respondents to select certain treatment modalities than the ones they would usually use in practice. Vignettes as a proxy measure for actual clinical behavior has been found to be a valid tool to measure clinicians decision making [36] and assessing quality of clinical practice [37]. A review by Hrisos et al. [38] found that clinicians self-reported performance on vignettes were, overall, close to performance observed by direct measure.

Closed-end checklists may result in a cueing effect, for respondents, to select additional actions. The different responses to the two identical closed-ended checklists for chronic and acute patients respectively, however indicate that this might not have been as big an issue, or at least that our respondents actually are aware that they treat chronic and acute patients differently.

### Perspectives

Danish chiropractors wishing to be considered experts in the field of musculoskeletal care should aim to incorporate clinical guidelines into the routine management and keep up to date with the newest research. Even though the Canadian Guidelines were not actively disseminated to Danish Chiropractors prior to this survey, this study shows low utilization of exercise therapy, especially with acute patients, which again results in low adherence to guideline recommendations for acute neck-pain patients in particular, but also for chronic neck-pain. More recent context specific Danish Guidelines [34] advocates for the use of exercise therapy for acute neck pain. As increased guideline compliance can lead to better patient care and health outcomes, higher patients satisfaction and reduce overall healthcare cost [7], decision makers and leaders in the Danish chiropractic profession should consider investing resources needed to increase guideline uptake by their members to improve patient care and health outcomes. For clinicians, this may imply spending less time on treatment modalities not supported by research and reallocating this time to prescribe exercise or delivering therapeutic exercise for acute neck pain patients. Future research should focus on Danish chiropractors’ compliance with the new Danish guidelines, and identifying effective knowledge translation strategies to increase the uptake of guidelines and evidence informed practice. There is a need for information on how to provide clear messaging to clinicians and patients on treatments supported by research.

## Conclusion

Our findings indicate that Danish chiropractors use a wide range of treatment options for managing adult patients with acute and chronic non-specific neck-pain. However, there were differences in treatments offered to acute and chronic patients, especially in regards to the use of exercise therapy, which were mainly reserved for chronic patients. Guideline compliance for acute and chronic neck-pain patients was low to moderate respectively, primarily because of underutilization of multimodal management. The low use of exercise therapy with acute patients contributed to this.

## Additional file

Additional file 1: Questionnaire. English version of the questionnaire used for data collection. (PDF 107 kb)
