# Supplementary material for: Managing patients with acute and chronic non-specific neck pain: are Danish chiropractors compliant with guidelines?
Source: Chiropr Man Therap. 2017 Jun 30;25:17. doi: 10.1186/s12998-017-0148-9 (PMC5492917; doi:10.1186/s12998-017-0148-9)
Supplement: Additional file 1: — Questionnaire. English version of the questionnaire used for data collection. (PDF 107 kb) [file 12998_2017_148_MOESM1_ESM.pdf]

Study on treatment of neck pain in chiropractic practice in Denmark

This questionnaire include questions about:

- Typical treatment modalities used in the clinic
- Considerations about choice of treatment modality
- Personal and demographic information

Do you currently work in a clinic or have you, during the last year, had clinical work with assesment and treatment of neck pain patients (including part-time)?

(1) ❑ Yes

(2) ❑ No

Part 1:

In this part of the questionnaire, you will be presented with different treatment modalities and we will ask you to answer how often you use the different modalities in your work with **treatment of patients with neck pain**. This applies to all treatment modalities you have, but also if you refer patients to other health care providers for this form of treatment.

1.1 Manipulation techniques (High-Velocity Low-Amplitude etc.)

Remember that we ask regarding neck pain patients!

	Often	Occasionally	Rarely	Never	Refer	Outside scope of practice
Diversified	(1) ❑	(2) ❑	(3) ❑	(4) ❑	(5) ❑	(6) ❑
Gonstead	(1) ❑	(2) ❑	(3) ❑	(4) ❑	(5) ❑	(6) ❑
Drop-techniques	(1) ❑	(2) ❑	(3) ❑	(4) ❑	(5) ❑	(6) ❑
Toggle	(1) ❑	(2) ❑	(3) ❑	(4) ❑	(5) ❑	(6) ❑
Activator	(1) ❑	(2) ❑	(3) ❑	(4) ❑	(5) ❑	(6) ❑
Other HVLA techniques	(1) ❑	(2) ❑	(3) ❑	(4) ❑	(5) ❑	(6) ❑

1.2 Region of manipulation

Remember that we ask regarding neck pain patients!

	Often	Occasionall y	Rarely	Never	Refer	Outside scope of practice
Segment of restriction / pain	(1) ❑	(2) ❑	(3) ❑	(4) ❑	(5) ❑	(6) ❑
Upper cervical	(1) ❑	(2) ❑	(3) ❑	(4) ❑	(5) ❑	(6) ❑
Thoracic	(1) ❑	(2) ❑	(3) ❑	(4) ❑	(5) ❑	(6) ❑
Lumbar	(1) ❑	(2) ❑	(3) ❑	(4) ❑	(5) ❑	(6) ❑
Pelvis	(1) ❑	(2) ❑	(3) ❑	(4) ❑	(5) ❑	(6) ❑
Extremity	(1) ❑	(2) ❑	(3) ❑	(4) ❑	(5) ❑	(6) ❑

1.3 Other manual therapy (done by you)

Remember that we ask regarding neck pain patients!

	Often	Occasionall y	Rarely	Never	Refer	Outside scope of practice
Triggerpoint (TrP)	(1) ❑	(2) ❑	(3) ❑	(4) ❑	(5) ❑	(6) ❑
Instrument Assisted Mobilization (IAM)	(1) ❑	(2) ❑	(3) ❑	(4) ❑	(5) ❑	(6) ❑
Massage	(1) ❑	(2) ❑	(3) ❑	(4) ❑	(5) ❑	(6) ❑
Massage device	(1) ❑	(2) ❑	(3) ❑	(4) ❑	(5) ❑	(6) ❑
Mechanical Diagnosis Therapy (MDT)/McKenzie	(1) ❑	(2) ❑	(3) ❑	(4) ❑	(5) ❑	(6) ❑
Mulligan Concept	(1) ❑	(2) ❑	(3) ❑	(4) ❑	(5) ❑	(6) ❑
Stretch/Muscle energy technique (MET)	(1) ❑	(2) ❑	(3) ❑	(4) ❑	(5) ❑	(6) ❑
Traction	(1) ❑	(2) ❑	(3) ❑	(4) ❑	(5) ❑	(6) ❑
Sacro Occipital Technique (SOT)	(1) ❑	(2) ❑	(3) ❑	(4) ❑	(5) ❑	(6) ❑
Cranio sacral technique	(1) ❑	(2) ❑	(3) ❑	(4) ❑	(5) ❑	(6) ❑

1.4 Other treatment modalities

Remember that we ask regarding neck pain patients!

	Often	Occasionally	Rarely	Never	Refer	Outside scope of practice
Transcutan Electric Nerve Stimulation (TENS)	(1) ❑	(2) ❑	(3) ❑	(4) ❑	(5) ❑	(6) ❑
Electromyography (EMG)	(1) ❑	(2) ❑	(3) ❑	(4) ❑	(5) ❑	(6) ❑
Short wave	(1) ❑	(2) ❑	(3) ❑	(4) ❑	(5) ❑	(6) ❑
Laser	(1) ❑	(2) ❑	(3) ❑	(4) ❑	(5) ❑	(6) ❑
Sonic	(1) ❑	(2) ❑	(3) ❑	(4) ❑	(5) ❑	(6) ❑
Ultrasound	(1) ❑	(2) ❑	(3) ❑	(4) ❑	(5) ❑	(6) ❑
Extracorporeal Shockwave Therapy (ESWT)	(1) ❑	(2) ❑	(3) ❑	(4) ❑	(5) ❑	(6) ❑
Heat (in clinic or home)	(1) ❑	(2) ❑	(3) ❑	(4) ❑	(5) ❑	(6) ❑
Cold (in clinic or home)	(1) ❑	(2) ❑	(3) ❑	(4) ❑	(5) ❑	(6) ❑
Acupuncture	(1) ❑	(2) ❑	(3) ❑	(4) ❑	(5) ❑	(6) ❑
Dry Needling (DN)	(1) ❑	(2) ❑	(3) ❑	(4) ❑	(5) ❑	(6) ❑

1.5 Training/Exercise (done by the patient)

Remember that we ask regarding neck pain patients!

	Often	Occasionally	Rarely	Never	Refer	Outside scope of practice
Stretch/MET cervical and upper thoracic region	(1) ❑	(2) ❑	(3) ❑	(4) ❑	(5) ❑	(6) ❑
Stretch/MET other body part	(1) ❑	(2) ❑	(3) ❑	(4) ❑	(5) ❑	(6) ❑
Strength cervical and upper thoracic region	(1) ❑	(2) ❑	(3) ❑	(4) ❑	(5) ❑	(6) ❑
Strength other body part	(1) ❑	(2) ❑	(3) ❑	(4) ❑	(5) ❑	(6) ❑
Motor control	(1) ❑	(2) ❑	(3) ❑	(4) ❑	(5) ❑	(6) ❑
Stability	(1) ❑	(2) ❑	(3) ❑	(4) ❑	(5) ❑	(6) ❑
Cardio-vascular	(1) ❑	(2) ❑	(3) ❑	(4) ❑	(5) ❑	(6) ❑
MDT/McKenzie	(1) ❑	(2) ❑	(3) ❑	(4) ❑	(5) ❑	(6) ❑
General physical activity	(1) ❑	(2) ❑	(3) ❑	(4) ❑	(5) ❑	(6) ❑

1.6 Orthoses/Assistive devices

Remember that we ask regarding neck pain patients!

	Often	Occasionally	Rarely	Never	Refer	Outside scope of practice
Collar	(1) ❑	(2) ❑	(3) ❑	(4) ❑	(5) ❑	(6) ❑
Pillow	(1) ❑	(2) ❑	(3) ❑	(4) ❑	(5) ❑	(6) ❑
Tape	(1) ❑	(2) ❑	(3) ❑	(4) ❑	(5) ❑	(6) ❑
Inserts	(1) ❑	(2) ❑	(3) ❑	(4) ❑	(5) ❑	(6) ❑
Other assistive devices	(1) ❑	(2) ❑	(3) ❑	(4) ❑	(5) ❑	(6) ❑

1.7 Information and guidance / patient education

Remember that we ask regarding neck pain patients!

	Often	Occasionally	Rarely	Never	Refer	Outside scope of practice
Diagnosis	(1) ❑	(2) ❑	(3) ❑	(4) ❑	(5) ❑	(6) ❑
Sideeffects	(1) ❑	(2) ❑	(3) ❑	(4) ❑	(5) ❑	(6) ❑
Monitor symptoms	(1) ❑	(2) ❑	(3) ❑	(4) ❑	(5) ❑	(6) ❑
Rest/offload	(1) ❑	(2) ❑	(3) ❑	(4) ❑	(5) ❑	(6) ❑
Active lifestyle	(1) ❑	(2) ❑	(3) ❑	(4) ❑	(5) ❑	(6) ❑
Diet and smoking	(1) ❑	(2) ❑	(3) ❑	(4) ❑	(5) ❑	(6) ❑
Supplements	(1) ❑	(2) ❑	(3) ❑	(4) ❑	(5) ❑	(6) ❑
Ergonomics	(1) ❑	(2) ❑	(3) ❑	(4) ❑	(5) ❑	(6) ❑
Work/job function	(1) ❑	(2) ❑	(3) ❑	(4) ❑	(5) ❑	(6) ❑
Free time	(1) ❑	(2) ❑	(3) ❑	(4) ❑	(5) ❑	(6) ❑
Employer communication	(1) ❑	(2) ❑	(3) ❑	(4) ❑	(5) ❑	(6) ❑
GP communication	(1) ❑	(2) ❑	(3) ❑	(4) ❑	(5) ❑	(6) ❑

1.8 Do you use other treatment modalities than those previously listed?

Remember that we ask regarding neck pain patients!

(1) ❑ Yes (please elaborate) _____

(3) ❑ No

Part 2:

In this next part, you will be presented with two short baseline cases. We will ask you to decide which groups of treatment modalities you will use in each case. We are thinking about a typical treatment course, not just at first visit.

Next up you will be presented with different additional findings / information and we will ask you to decide whether or not these could get you to change your treatment strategy

Case 1 (acute)

A 39-year-old man addresses you with emerging local neck pain through 2 days, occurred with no known cause. There are no other musculoskeletal complaints. He has no other health problems now or earlier. There are no red flags. He exercise regularly and seems to be in good shape both physically and mentally. There is no physical function limitation at work or at home. No previous neck pain.
Objectively you find local pain and restricted movement of the neck.

There is no immediate indication for X-rays.

2.1 How would you treat this patient?

	Often	Occasionally	Rarely	Never	Refer	Outside scope of practice
Manipulation techniques	(1) ❑	(2) ❑	(3) ❑	(4) ❑	(5) ❑	(6) ❑
Other manual therapy	(1) ❑	(2) ❑	(3) ❑	(4) ❑	(5) ❑	(6) ❑
Other treatment modalites	(1) ❑	(2) ❑	(3) ❑	(4) ❑	(5) ❑	(6) ❑
Training/Exercise	(1) ❑	(2) ❑	(3) ❑	(4) ❑	(5) ❑	(6) ❑
Orthoses/assistive devices	(1) ❑	(2) ❑	(3) ❑	(4) ❑	(5) ❑	(6) ❑
Information and guidance	(1) ❑	(2) ❑	(3) ❑	(4) ❑	(5) ❑	(6) ❑

Case 1 (acute)

Same as previous

A 39-year-old man addresses you with emerging local neck pain through 2 days, occurred with no known cause. There are no other musculoskeletal complaints. He has no other health problems now or earlier. There are no red flags. He exercise regularly and seems to be in good

shape both physically and mentally. There is no physical function limitation at work or at home. No previous neck pain.
Objectively you find local pain and restricted movement of the neck.

There is no immediate indication for X-rays.

2.2 How likely is it that you would change your treatment strategy if:

	Very likely	Likely	Unlikely	Very unlikely	Dont know
The patient also indicated significant functional impairment and 10/10 on the pain scale	(1) ❑	(2) ❑	(3) ❑	(4) ❑	(5) ❑
The patient also suffered from depression / stress / anxiety	(1) ❑	(2) ❑	(3) ❑	(4) ❑	(5) ❑
The patient instead was significantly overweight and did not regular exercise	(1) ❑	(2) ❑	(3) ❑	(4) ❑	(5) ❑
The patient had signs of nerve root involvement	(1) ❑	(2) ❑	(3) ❑	(4) ❑	(5) ❑
The patient had previously experienced pain episodes	(1) ❑	(2) ❑	(3) ❑	(4) ❑	(5) ❑
The symptoms were caused by a traffic accident and subsequent imaging was blank	(1) ❑	(2) ❑	(3) ❑	(4) ❑	(5) ❑

Case 2 (chronic)

A 57-year-old woman contacts you with lengthy constant neck pain during the last 5-7 years, occurred with no known cause. There are no other musculoskeletal complaints. Indicates no other health problems now or earlier. There are no red flags. She exercise regularly and seems to be in good shape both physically and mentally. There is no physical function limitation at work or at home. Have tried treatment by physiotherapist with only short-term relief.
Objectively you find local pain and restricted movement of the neck.
Radiographs show age-related mild degenerative changes

2.3 How would you treat this patient?

	Often	Occasionally	Rarely	Never	Refer	Outside scope of practice
Manipulation techniques	(1) ❑	(2) ❑	(3) ❑	(4) ❑	(5) ❑	(6) ❑
Other manual therapy	(1) ❑	(2) ❑	(3) ❑	(4) ❑	(5) ❑	(6) ❑
Other treatment modalites	(1) ❑	(2) ❑	(3) ❑	(4) ❑	(5) ❑	(6) ❑
Training/Exercise	(1) ❑	(2) ❑	(3) ❑	(4) ❑	(5) ❑	(6) ❑
Orthoses/assistive devices	(1) ❑	(2) ❑	(3) ❑	(4) ❑	(5) ❑	(6) ❑
Information and guidance	(1) ❑	(2) ❑	(3) ❑	(4) ❑	(5) ❑	(6) ❑

Case 2 (chronic)

Same as previous

A 57-year-old woman contacts you with lengthy constant neck pain during the last 5-7 years, occurred with no known cause. There are no other musculoskeletal complaints. Indicates no other health problems now or earlier. There are no red flags. She exercise regularly and seems to be in good shape both physically and mentally. There is no physical function limitation at work or at home. Have tried treatment by physiotherapist with only short-term relief.
Objectively you find local pain and restricted movement of the neck.
Radiographs show age-related mild degenerative changes

2.4 How likely is it that you would change your treatment strategy if:

	Very likely	Likely	Unlikely	Very unlikely	Dont know
The patient also indicated significant functional impairment and 10/10 on the pain scale	(1) ❑	(2) ❑	(3) ❑	(4) ❑	(5) ❑
The patient also suffered from depression / stress / anxiety	(1) ❑	(2) ❑	(3) ❑	(4) ❑	(5) ❑
The patient instead was significantly overweight and did not regular exercise	(1) ❑	(2) ❑	(3) ❑	(4) ❑	(5) ❑
The patient had signs of nerve root involvement	(1) ❑	(2) ❑	(3) ❑	(4) ❑	(5) ❑
The patient also indicated significant functional	(1) ❑	(2) ❑	(3) ❑	(4) ❑	(5) ❑

	Very likely	Likely	Unlikely	Very unlikely	Dont know
impairment and 10/10 on the pain scale					
The patients ability to work was threatned	(1) ❑	(2) ❑	(3) ❑	(4) ❑	(5) ❑
The patient also had an unresolved case about disability pension	(1) ❑	(2) ❑	(3) ❑	(4) ❑	(5) ❑

Part 3:

In this last part of the questionnaire asks about personal and demographic information.
Please tick the most appropriate answer.

3.1 Sex

(1) ❑ Male
(2) ❑ Female

3.2 Age

_____

3.3 Year of graduation

_____

3.4 Country of graduation

(0) ❑ Denmark
(1) ❑ United Kingdom
(2) ❑ United States of America
(3) ❑ Canada
(4) ❑ Other _____

3.5 What are you? (possible to tick more than one)

(0) ❑ Clinic owner

(1) ❑ Private employee

(2) ❑ Public employee

(3) ❑ Health ensurance employee

(4) ❑ Other: _____

3.6 In which region(s) do you have your clinical work? (possible to tick more than one)

(0) ❑ North Denmark Region

(1) ❑ Central Denmark Region

(2) ❑ Region of Southern Denmark

(3) ❑ Region Zealand

(4) ❑ Capitol region of Denmark

The questionnaire is over, thank you for taking the time to reply
